# Co-existence of overweight/obesity and stunting: it’s prevalence and associated factors among under - five children in Addis Ababa, Ethiopia

**DOI:** 10.1186/s12887-022-03445-5

**Published:** 2022-06-29

**Authors:** Alem Sebsbie, Abebe Minda, Sindew Ahmed

**Affiliations:** 1 Department of Public Health Kotebemetropolitan University, Deberbirhan, Ethiopia; 2 Department of Public Health Deberbirhan University, Deberbirhan, Ethiopia; 3 Department of Nursing Kotebemetropolitan University, Deberbirhan, Ethiopia

**Keywords:** Stunting, Obesity, Children, Ethiopia

## Abstract

**Background:**

Double burden of malnutrition is a global problem posing a serious public health challenge especially in low- and middle-income countries including Ethiopia, where a high prevalence of under-nutrition continues to exist and overweight is increasing at an alarming rate. Although both under-nutrition and over-nutrition are investigated extensively in Ethiopia, evidence about the double burden of malnutrition especially at the individual level is very limited.

**Objective:**

To assess the prevalence of the co-existence of overweight/obesity and stunting and associated factors among under-five children in Addis Ababa, Ethiopia at an individual level.

**Methods:**

Institution-based cross-sectional study was conducted from May to June 2021 among 422 mothers to child pairs in Addis Ababa. Twenty-nine (30%) of the health centers in Addis Ababa were selected to take part in the study using a simple random sampling technique. The total sample size was allocated proportionally to each of the selected health centers based on their performances within 6 months prior to the study. A systematic random sampling method was used to select the study participants. An interviewer-administered structured questionnaire was used to collect data. Descriptive statistics and a hierarchical logistic regression model were used to characterize the study population and to identify factors that are associated with the outcome variable respectively. Odds ratio along with 95% CI were estimated to measure the strength of the association. The level of statistical significance was declared at a *p*-value less than 0.05.

**Results:**

The prevalence of the co-existence of overweight/obesity and stunting was 5.1% with 95% CI (2.9–7.1%). The hierarchical logistic regression analysis revealed that child age (6–23 months) [(AOR = 2.86, 95% CI: (1.02–8.04)], maternal education status (non-educated) [(AOR = 4.98, 95% CI: (1.33–18.66)], maternal age during birth (≥ 28 years) [(AOR = 0.22, 95% CI: (0.06–0.79)] and childbirth order (3+) [(AOR = 6.38, 95% CI: (1.03–39.7)] were significantly associated with the co-existence of overweight /obesity and stunting. Conclusion and recommendations: The study revealed that the prevalence of the co-existence of overweight/obesity and stunting is low in Ethiopia. However, local and national nutrition policies and programs should be tailored and implemented to simultaneously address both under-nutrition and over-nutrition.

**Supplementary Information:**

The online version contains supplementary material available at 10.1186/s12887-022-03445-5.

## Introduction

The double burden of malnutrition (DBM) is defined as the co-occurrence of under-nutrition along with over-nutrition or diet related non communicable diseases (NCDs) [1–4]. This co-occurrence or simultaneous existence of under-nutrition (predominately stunting) and over-nutrition (overweight/obesity) is also termed as nutritional dual-burden [5]. It can occur at population, household and individual levels [5–7].

The DBM is a global problem posing a serious public health challenge especially in low- and middle-income countries (LMIC), where high prevalence of under-nutrition continues to exist and overweight is increasing at an alarming rate [1, 8–11].

According to the global nutrition report for the year 2018, the prevalence of the co-existence of overweight and stunting among under-five children is 1.87% (8.23 million) globally. The magnitude of the coexistence of overweight/obesity and stunting is 2.7% in Europe, 2.3% in Africa and 0.8% in the Americas [12].

Evidence have indicated that DBM is more prevalent in urban areas and it is a concern particularly for countries having a high prevalence rate of stunting [13, 14]. It particularly affects the urban poor, the rural rich and people living in slum areas [15]. Children aged below 5 years are also the most susceptible age group to DBM [6].

Sub-Saharan Africa (sSA) is suffering with the DBM with high magnitude of under-nutrition and an increasing burden of overweight/obesity and diet-related NCDs [16]. Ethiopia is not an exception since, the country is undergoing nutrition transition as a result of economic growth and urbanization, which could led to rise in the magnitude of DBM [17]. In Ethiopia, malnutrition has been declining over the last two decades as a result of the implementation of both nutrition specific interventions and nutrition sensitive interventions guided by the National Nutrition Programs (NNP I & NNP II) [18]. However, the problem of under nutrition particularly stunting remains as a major public health problem in Ethiopia and simultaneously the magnitude of overweight and obesity is increasing rapidly especially in urban areas [19–21]. The problem of childhood obesity in Ethiopia is not recognized as a serious problem and lacks adequate attention [22].

The study setting, Addis Ababa, is the capital city of Ethiopia and it is the biggest and most rapidly growing city where 25% of the country’s urban population lives [23]. Although the prevalence of stunting is lower in Addis Ababa compared to the other regions of Ethiopia, still 19.6% of under-five children are suffering with stunting and 11.4% with overweight/obesity [24]. Evidence regarding DBM in Ethiopia is very scarce especially at individual level and particularly in children. Most of the studies are concentrated in investigating under-nutrition and overweight/obesity independently. Therefore, this study was aimed to assess the co-existence of overweight/obesity and stunting and associated factors among children aged 6–59 months.

## Methods

### Study area

The research was carried out in Addis Ababa, Ethiopia’s capital and largest city. Addis Ababa is a chartered city with three levels of authority: city government at the top, 11 sub-cities in the middle, and 126 woredas at the bottom. The total population of the city for the year 2020 was estimated to be 4,793,699 [25]. According to Addis Ababa health bureau, the number of under-five children in 2021 was 342,989 and 304,879 (6.4%) of them were children between 6 and 59 months of age. In the city, six governmental hospitals and 98 health centers are providing comprehensive health care services to the population of the city.

### Study design and period

An institution based cross-sectional study design was conducted from May to June, 2021.

### Study population

All children aged 6 to 59 months with their respective mothers/care givers who were residing in Addis Ababa, Ethiopia was the source population. The study population was all randomly selected children aged 6 to 59 months with their respective mothers/caregivers who visited public health centers for growth monitoring and promotion services, vaccination services, Vitamin A supplementation, deworming and under-five outpatient department (OPD) in Addis Ababa during the study period.

### Sample size determination

Sample size was determined based on a single population proportion formula assuming, proportion (*P* = 50%) because of lack of evidence in Ethiopia, and to get maximum sample size, confidence level (95%) and margin of error (5%), the minimum required sample size was 384. Adding 10% for non-response rate, the final sample size was 422.

### Sampling procedure

Simple random sampling method was employed among 98 health centers to select 29 (30%) of the health centers. Then the final sample size was allocated proportionally to each of randomly selected health centers based on their performances (daily average number of under-five children who have been coming to the health center seeking health care services). Systematic sampling method was used to select study participants (mother to child pairs) from each of randomly selected health centers within the predetermined study period.

### Data collection procedures

Data were collected using interviewer administered questionnaire which was adapted from various similar studies [17, 26–32] from mothers /caregivers of children aged 6 to 59 months. Six B.Sc. holder health professionals (four data collectors and two supervisors) who had experience in data collection and supervision were recruited and deployed to collect data and supervise the process. Prior to data collection, the data collectors and supervisors received 3 days theoretical and practical training on the study.

### Anthropometric measurement

#### Height

Height/length measurements were carried out with standard measuring boards to the nearest 0.1 cm. Children under the age of 24 months were measured in lying down (recumbent) position on the board, while children aged 24 to 59 months were measured in a standing-up position. Mothers were requested to remove their children’s shoes, hair ornaments and other things that interfere in the measurement of the length/height of the child [33].

#### Weight

Weight of infants was measured using a Salter spring scale while young children using digital beam balance with a minimum cloth and barefoot to the nearest of 0.1 kg. Weighting scales were calibrated regularly. Height for age Z score (HAZ) and weight for height Z score (WHZ) were determined using WHO Anthro software version 3.1.0.

#### Wealth index

Wealth index was calculated using principal component analysis (PCA). Mothers were asked questions about their household fixed assets and housing condition adapted from the Ethiopian demographic and health survey report (EDHS-2016) [34].

### Data management analysis

Data were entered in to Epi-Info version 7.2 Software. Then data were exported into statistical software package for social sciences (SPSS) version 20 for analysis after performing data cleaning. Descriptive statistics were computed to summarize and describe the data. Binary logistic regression model was fitted to identify factors associated with the outcome variable. Variables with the result of *p*-value of less than 0.25 in the bi-variable analysis were entered in to the multivariable analysis (hierarchical logistic regression model). Crude odds ratio (COR) and adjusted odds ratio (AOR) using 95% confidence interval were computed to see the strength of associations. A p – value of less than 0.05 in the hierarchical logistic regression analysis was used to declare statistical significance.

### Data quality management

To ensure data quality, experienced data collectors and supervisors were recruited, deployed and trained. Furthermore, pre-testing of the questionnaire was carried out. The data collectors were also supervised and provided onsite technical assistance both by the supervisors and the principal investigator to assure the quality of data. In addition to this, data completeness and consistency were checked on daily basis and corrective measures were taken timely. Moreover, measurement equipment was calibrated regularly before starting the anthropometric measurements. After data collection, each questionnaire was coded and checked for completeness and consistency prior to data entry. Checking of data for missed values, inconsistencies and outliers were also done after data entry in to EPI-Info version 7.2 and after exporting into SPPS version 20.

### Variable measurement

#### Outcome variable

The outcome variable was co-existence of overweight/obesity and stunting which is defined as the existence of both overweight/obesity (WHZ score > + 2 SD) and stunting (HAZ score of < − 2 SD) with in the same child. It was dichotomized in to co-existence of overweight/obesity and stunting as “Yes” or “No”.

#### Exposure variables

The predictor variables were categorized into child characteristics, distal factors, intermediate factors and proximal factors. The child characteristics include child age and child sex. The distal factors were maternal education, maternal occupation, father’s education, and head of the household and house hold wealth index category.

The Intermediate factors were marital status; family size, number of under-5 children, maternal age at child birth, and type of family, availability of health insurance, child birth order, and child ever received any vaccinations and type of latrine.

The proximal factors were weight of the child at birth, child ever breast fed, time of initiation of breast feeding, duration of breast feeding, age initiated for complementary feeding (CF), diarrhea in the previous 2 weeks, cough in the previous 2 weeks, fever in the previous 2 weeks, vitamin A dose supplementation within last 6 months, and child dewormed within the last 6 months.

##### Ethical consideration

Ethical approval were obtained from Ethical review committee of Kotebe metropolitan University, Menelik II Medical and Health Science College and Addis Ababa public health Research and Emergency management directorate After the research proposal was duly reviewed by Addis Ababa health Bureau IRB. The researchers were informed with copy of letter to report any change in the study procedure and submit an activity progress report to the Ethical commute as required. Then permission letter were obtain from Addis Ababa education Bureau and selected primary school. “Informed consent” was obtained from selected student’s parent, after clearly informed about; the purpose of the study. Parents were inform they could withdraw from the participation at any time. Privacy and confidentiality of information taken from respondent keep properly and names was not record. The Author also declared that all methods were carried out in accordance with relevant guidelines and regulations.

## Results

A total of 411 mothers and children were participated in the study with a response rate of 97.4%.

### Socio - demographic characteristics of respondents

The mean (± SD) age of the children was 2.28 years with (± 1.18). Two hundred thirty (56%) of the children were male. The majority (87.4%) and (46%) of the mothers attended formal education and were housewives respectively (Table 1).

**Table 1:** Socio - demographic characteristics of respondents, Addis Ababa, Ethiopia 2021

Variables	Category	Frequency	Percent
Child sex	Male	230	56
	Female	181	44
Child age in months	6–11	63	15.3
	12–23	124	30.2
	24–35	109	26.5
	36–47	72	17.5
	48–59	43	10.5
Maternal education	No education	52	12.6
	Primary school	115	28.0
	Secondary school	108	26.3
	Diploma	82	20.0
	Degree & above	54	13.1
Maternal occupation	Housewife	189	46.0
	Government employee	82	20.0
	Merchant	31	7.5
	Non-government employee	36	8.8
	Self-employee	70	17
	Others	3	**0.7**
Fathers education	No education	20	4.8
	Primary school	57	13.9
	Secondary school	168	40.9
	Diploma	71	17.3
	Degree & above	95	23.1
Head of the household	Father	196	47.7
	Mother	18	4.4
	Both	197	47.9
Wealth index	Lowest	83	20.2
	Second	82	20.0
	Middle	82	19.9
	Fourth	85	20.7
	Highest	79	19.2

### Magnitude of the co-existence of overweight/obesity and stunting

**Fig. 1:**
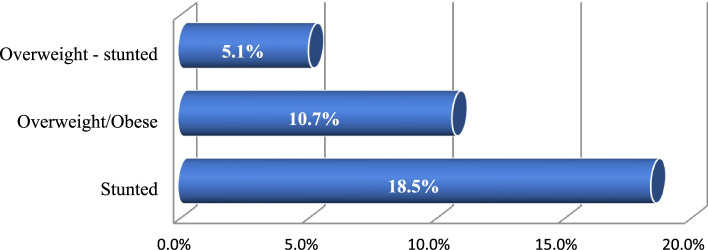
Magnitude of the co-existence of overweight/obesity and stunting among under-five children in Addis Ababa, Ethiopia, 2021

According to the study, the magnitude of the co-existence of overweight/obesity and stunting was 5.1% with 95% CI (2.9–7.1%) (Fig. 1).

### Intermediate factors associated with the co-existence of overweight/obesity and stunting

#### Environmental factors associated with the co-existence of overweight/obesity and stunting

Half (50.4%) of the respondents had improved sanitation facility. Most (79.3%) of under – five children participated in the study were living with nuclear family. Almost two-third (64.7%) of the respondents had a family size of 4 and above (Table 2).

**Table 2:** Environmental factors associated with the co-existence of overweight/obesity and stunting in under-five children in Addis Ababa, Ethiopia 2021

Variables	Category	Frequency	Percent
Type of latrine	Improved	207	50.4
	Unimproved	204	49.6
Type of family	Nuclear family	326	79.3
	Extended family	85	20.7
Family size	< 4	145	35.3
	≥ 4	266	64.7
No of under – five children in the HH	1	274	66.7
	2	125	30.4
	3^+^	12	2.9

### Maternal factors associated with the co-existence of overweight/obesity and stunting

The majority (92.5%) and (64.7%) of the respondents were married and in age group between 20 to 29 years. Only 123 (30%) of the respondents had health insurance at household level (Table 3).

**Table 3:** Maternal factors associated with the co-existence of overweight/obesity and stunting among under-five children in Addis Ababa, Ethiopia 2021

Variables	Category	Frequency	Percent
Marital status	Married	380	92.5
	Single	11	2.7
	Separated	10	2.4
	Widowed	5	1.2
	Divorced	5	1.2
Maternal age at child birth	< 20	15	3.7
	20–29	266	64.7
	30–39	130	31.6
Birth order of the child	1	189	46.0
	2	159	38.7
	3^+^	63	15.3
Child ever Vaccinated	Yes	411	100
	No	0	–
Immunization status of the child	Fully immunized	251	61.1
	Currently on follow up	146	35.5
	Not fully immunized	14	3.4
Availability of Health insurance at HH level	Yes	123	29.9
	No	288	70.1

### Proximal factors associated with the co-existence of overweight/obesity and stunting

#### Individual level factors associated with the co-existence of overweight/obesity and stunting

Three hundred fifty four (86.1%) children had average birth weight (2.5–4 kg). More than half (53.3%) of the respondents had breast fed their child for 12–24 months. Two hundred fifty nine (63%) of the mothers started complementary feeding at 6 month for their children. Only 183 (44.5%) of children had dewormed in the previous 6 months prior to the data collection (Table 4).

**Table 4:** Proximal factors associated with the co-existence of overweight/obesity and stunting among under - five children in Addis Ababa, Ethiopia 2021

Variables	Category	Frequency	Percent
Birth weight	< 2.5 kg	35	8.5
	2.5–4 kg	354	86.1
	> 4 kg	22	5.4
Child ever been breastfed	Yes	405	98.5
	No	6	1.5
Time of initiation of breast feeding	Within one hour	284	69.1
	Within the first 24 hours	102	24.8
	> 24 hours	25	6.1
Duration of breast feeding	< 12 months	147	35.8
	12–24 months	219	53.3
	> 24 months	45	10.9
Age initiated for CF	Before 6 months	123	29.9
	At 6 month	259	63.0
	After 6 months	29	7.1
Diarrhea in the previous 2 weeks	Yes	74	18.0
	No	337	82.0
Cough in the previous 2 weeks	Yes	82	20.0
	No	329	80.0
Fever in the previous 2 weeks	Yes	94	22.9
	No	317	77.1
Vitamin A supplement	Yes	337	82.0
	No	74	18.0
Deworming	Yes	183	44.5
	No	228	55.5

### Factors associated with the co-existence of overweight/obesity and stunting

#### Results of bi-variable analysis

Thirteen variables, child age, maternal education, maternal occupation and level of father’s education among the distal factors, family size, maternal age at child birth, birth order and availability of health insurance from intermediate factors, and birth weight, duration of breast feeding, age initiated for complementary feeding, vitamin A supplement and deworming in the previous 6 months from the proximal factors had *p*-value less than 0.25 (*p* < 0.25) with the co-existence of overweight/obesity and stunting in the bi-variable analysis and were candidates for the multivariable analysis.

#### Results of multivariable analysis

Three step wise models in the hierarchical logistic regression analysis were used to identify factors associated with the co-existence of overweight/obesity and stunting. In model one distal factor were entered to estimate their effect on the outcome variable. Model two revealed the relationship among distal factors, intermediate factors and the co-existence of overweight/obesity and stunting. Model three was used to evaluate the combined effects of distal, intermediate and proximal factors on the outcome variable. Child characteristics (age and sex) are retained in all the three models.

In model one, child age [(AOR = 3.38, (95% CI: (1.26–9.09)] and maternal education [(AOR = 5.73, 95% CI: (1.78–18.43)] were significantly associated with the co-existence of overweight/obesity and stunting. In model two (after the addition of intermediate factors) child age [(AOR = 2.86, 95% CI: (1.02–8.04)], maternal education [(AOR = 5.18, 95% CI: (1.51–17.76)] and maternal age during birth [(AOR = 0.26, 95% CI: (0.08–0.87)] revealed significant association. In the final model (after the addition of proximal factors in model three), maternal education [(AOR = 4.98 95% CI: (1.33–18.66)], maternal age during birth [(AOR = 0.22, 95% CI: (0.06–0.79)] and birth order of the child [(AOR = 6.38, 95% CI: (1.03–39.7)] were significantly associated with the co-existence of overweight/obesity and stunting (Table 5).

**Table 5:** Bi-variable and multivariable analysis of factors associated with the co-existence of stunting and overweight/obesity among under- five children in Addis Ababa, Ethiopia 2021

Variables	CEOS		COR (95% CI)	AOR (95% CI)		
	Yes	No		Model 1	Model 2	Model 3
Child age in months						
6–23	15	172	3.17 (1.20, 8.34)*	3.38 (1.26, 9.09)*	2.86 (1.02, 8.04)*	1.69 (0.48, 6.05)
24–59	6	218	Ref.	Ref.	Ref.	Ref.
Maternal education						
Non – educated	7	45	3.83 (1.47, 10)*	5.73 (1.78, 18.43)*	5.18 (1.51, 17.76)*	4.98 (1.33, 18.66)*
Educated	14	345	Ref.	Ref.	Ref.	Ref.
Birth order						
1	14	175	Ref.		Ref.	Ref.
2	3	156	0.240 (068, 0.852)*	NA	0.98 (0.18, 5.31)	0.89 (0.16, 5.03)
3^+^	4	59	0.847 (0.268, 2.68)	NA	4.82 (0.84, 27.82)	6.38 (1.03, 39.7)*
Maternal age during birth						
< 28	16	178	Ref.		Ref.	Ref.
≥ 28	5	212	0.262 (0.09, 0.73)*	NA	0.26 (0.08, 0.87)*	0.22 (0.06, 0.79)*

*Ref.* Reference

*NA* Not applicable

* - Significant at *p* value < 0.05

## Discussion

The study revealed that magnitude of the co-existence of overweight/obesity and stunting among under-five children was 5.1%: showing that Ethiopia is experiencing the double burden of malnutrition at individual level. The finding is comparable with a study conducted in Mexico (5%) and India (5.4%) [29, 35]. But it is higher than studies conducted in Kenya (1%), South Africa (1.2%), Vietnam (1.4%), Bolivia (2.3%), Thailand (1.3%) and Colombia (0.1%) [13, 27, 28, 36–38]. The possible reason for this might be high prevalence rate of stunting in Ethiopia [19]. This is because countries having higher magnitude of under-nutrition are more at risk for an increased prevalence of obesity [31, 39]. However the result is lower than findings from Egypt (10.9%), Ghana (19%) and Mexico (10.3%) [8, 29, 32]. The possible reason for this may be the difference in socio-economic status, urbanization and the stage of nutrition transition among countries. The difference in study period and sample size might be also another possible reasons for the discrepancies in the prevalence of CEOS across countries.

The study showed that child age is significantly associated with the co-existence of overweight/obesity and stunting in model two. The odd of the co-existence of overweight/obesity and stunting among children aged 6–23 months was 2.86 times higher than that of children aged 24–59 months. This finding is in line with a study conducted in Indonesia and Papua New Guinea [31, 40]. The possible reason could be due to feeding practices of the mother/caretaker within the first 1000 days of life. Malnutrition in children is associated with poor breastfeeding practice and inappropriate offering of solid foods [41]. This is explained by the fact that inappropriate offering of solid food and poor breast-feeding practice will cause stunting because of infection as of breast feeding is a means to boost the immunity of the child and the other hand it may be leads to an intentional weight gain. That is why children aged under 3 years are most at risk for stunting, which may be associated with increased risk of being overweight in later life [32].

The study also noted that maternal education status was strongly associated with the co-existence of overweight/obesity and stunting in the final model. The odd of the co-existence of overweight/obesity and stunting among children belonging to non-educated mothers was 4.98 times higher than that of children belonging to educated mothers. This result is in agreement with a study conducted in Cameroon [30], china [42] and Guatemala cited in Kosaka and Umezaki, 2017 [43]. This might be because women are often considered as primary caregivers [44] and therefore lack of knowledge and certain attitudinal factors by the mother could eventually influence nutritional status of children through feeding practices [45]. Having formal education also enables mothers to take better care, better utilization of the health services, and also to implement better hygiene practice of their child [46].

The other factor which is significantly associated with the co-existence of overweight/obesity and stunting in the final model is maternal age during birth of the child. Children whose mother had maternal age < 28 during birth were 78% more likely to experience the co-existence of overweight/obesity and stunting compared to mothers aged ≥28 during birth. This result is in agreement with a study conducted in Cameroon [30]. It is also supported by a finding from Mexico [29]. The possible explanation for this could be young maternal age during pregnancy is correlated with shorter newborn birth length and small for gestational age delivery [47].

In the final model, children of 3rd and above birth order were 6.38 times more likely to be affected by the co-existence of overweight/obesity and stunting compared to children of 1st birth order. This is supported by studies conducted in Bangladesh [48] and Sub Saharan Africa in 18 countries [49]. This is because children born later are vulnerable to sub-optimal nutrition and health outcomes [50]. The other explanation could be that as the number of births rises, the food and resources allocated to family members in a household decreases. Therefore, births of higher order might be affected by malnutrition and other health problems [48].

### Study limitations

The study was a facility based and as a result prevents generalization to all under five children living in Ethiopia. In addition to this, the study did not assess variables which could be potentially linked to the co-existence of overweight/obesity and stunting such as children’s physical activity, dietary diversity, maternal height and weight. Moreover, the study was also subjected to recall bias since some of the variables were dependent on the memory of mothers and might lead to recall bias.

### Strengths

- Hierarchical logistic regression model was used to identify factors that determine the co-existence of overweight/obesity and stunting independently

## Conclusion

The study revealed that the magnitude of the co-existence of overweight/obesity and stunting among under-five children in Addis Ababa was low. In addition to this, child age, maternal education, birth order of the child and maternal age during birth were found to be significantly associated with the co-existence of overweight/obesity and stunting. Therefore, access to formal education for females should be improved. Maternal health programs should also emphasize on improving service uptake and quality of family planning services to delay early pregnancy and reduce the number of high birth order pregnancies. Further research using longitudinal study design and large sample size are also needed to understand the real contributors of the co-existence of overweight/obesity and stunting.

## Supplementary Information

**Additional file 1.**

## Data Availability

All data are available as a supplementary file.
